# The quantification of vaccine uptake in the Nordic countries and impact on key indicators of COVID-19 severity and healthcare stress level via age range comparative analysis

**DOI:** 10.1038/s41598-022-21055-0

**Published:** 2022-10-07

**Authors:** Anna Sigridur Islind, María Óskarsdóttir, Corentin Cot, Giacomo Cacciapaglia, Francesco Sannino

**Affiliations:** 1 Department of Computer Science, Reykjavík University, Menntavegur 1, 102 Reykjavík, Iceland; 2 Institut de Physique des deux Infinis de Lyon (IP2I), UMR5822, CNRS/IN2P3, 69622 Villeurbanne, France; 3 University of Lyon, Université Claude Bernard Lyon 1, 69001 Lyon, France; 4 CP3-Origins & the Danish Institute for Advanced Study, University of Southern Denmark, Campusvej 55, 5230 Odense, Denmark; 5 Dipartimento di Fisica E. Pancini, Università di Napoli Federico II & INFN sezione di Napoli, Complesso Universitario di Monte S. Angelo, Edificio 6, via Cintia, 80126 Naples, Italy

**Keywords:** Health care, Statistics, Applied mathematics

## Abstract

In this paper we analyze the impact of vaccinations on spread of the COVID-19 virus for different age groups. More specifically, we examine the deployment of vaccines in the Nordic countries in a comparative analysis where we focus on factors such as healthcare stress level and severity of disease through new infections, hospitalizations, intensive care unit (ICU) occupancy and deaths. Moreover, we analyze the impact of the various vaccine types, vaccination rate on the spread of the virus in each age group for Denmark, Finland, Iceland, Norway and Sweden from the start of the vaccination period in December 2020 until the end of September 2021. We perform a threefold analysis: (i) frequency analysis of infections and vaccine rates by age groups; (ii) rolling correlations between vaccination strategies, severity of COVID-19 and healthcare stress level and; (iii) we also employ the epidemic Renormalization Group (eRG) framework. The eRG is used to mathematically model wave structures, as well as the impact of vaccinations on wave dynamics. We further compare the Nordic countries with England. Our main results outline the quantification of the impact of the vaccination campaigns on age groups epidemiological data, across countries with high vaccine uptake. The data clearly shows that vaccines markedly reduce the number of new cases and the risk of serious illness.

## Introduction

In December 2019, a severe respiratory disease emerged in Wuhan, China^1^. Since the first identified case of COVID-19 was reported on 8. December 2019^2,3^, the virus has spread quickly, causing a worldwide health crisis. The spread has occurred in waves, and the virus evolves and mutates, and consequently we have yet not seen the final numbers of ebb and flow with 215 million confirmed cases and 4.48 million confirmed deaths as of end of September 2021 and 596 million confirmed and 6.45 million confirmed cases a year later, at the end of September 2022. To halt the spread, various non-pharmaceutical methods were put into place, such as limited travel in combination with social distancing, which proved to be important measures to slow down the short-term spread^4^. Eventually, pharmaceutical interventions were administered through vaccinations, to slow down the long-term spread^5,6^. The first vaccine doses were administered in December 2020, a year after the pandemic started. Since then, vaccinations have been seen as the utmost important strategy for containing the spread of the virus, for minimizing risks for citizens and ultimately for slowly alleviating the non-pharmaceutical restrictions that were put in place^6^. In September 2021, 33% of the world population had received at least one dose of a COVID-19 vaccine and 25.1% were fully vaccinated. As of September 2022, 63% of the world population were fully vaccinated.

The virus is particularly vigilant and a paper based on data from the European mortality monitoring activity network (EuroMOMO) concluded that all-cause excess mortality in Europe until April 2020, 90% of excess mortality was due to COVID-19 in the age group of 64 and older^7^, pointing towards the notion that age should outline the primary criteria for vaccination prioritisation. Other papers have showed that super-spreaders are the important link in taming the pandemic, as they spread the virus to the healthy and the ill; ergo they should be vaccinated first^8–10^. Since there are several different standpoints on the topic, it is important to quantify and compare the temporal impact of vaccinations, through a data-driven approach.

As of September 2021, a total of 623 million COVID-19 vaccine doses have been distributed within the European Union and European Economic Area (EU/EEA) and 530 million of those doses have already been given to citizens. In the EU/EEA, the cumulative vaccine uptake since the first doses were administered is 75.9% for first dose and 67.3% of citizens are fully vaccinated (see https://vaccinetracker.ecdc.europa.eu/public/extensions/COVID-19/vaccine-tracker.html). The time from vaccination start, until September 2021 was a critical time frame, and it is therefore interesting to retrospectively examine the data from that period. Furthermore, the vaccination uptake was and is especially high in some European countries. In the Nordic countries (Denmark, Finland, Iceland, Norway and Sweden), the vaccination uptake has been notably high. In Iceland 91.2% of the population had received one dose and 76.5% were fully vaccinated in September 2021 and a year later, 82% were fully vaccinated. In Denmark 88.6% of the population had received one dose and 71.5% was fully vaccinated in September 2021, and a year later fully vaccinated were 82.1%. In Finland 84.2% of the population had received one dose and 50.3% were fully vaccinated in September 2021 and a year later fully vaccinated were 78.5%. In Norway 87% of the population had received one dose and 56.6% is fully vaccinated and a year later fully vaccinated were 75.3%. In Sweden 81.7% of the population had received one dose and 55.6% were fully vaccinated and a year later the fully vaccinated were 74%. Likewise, England had high numbers early. Since the vaccine uptake was high already in September 2021, we rely on data up until that point in our analysis.

Due to limited supply of vaccine, and because the virus is highly contagious, the way the vaccine for COVID-19 is prioritized in each country, is utmost important^11^. The strategies for prioritization vary between countries, where some countries administer the vaccine first to those of higher age or with serious health issues while other countries prioritize those in high-exposure occupations which are considered a greater risk for spreading the virus^12,13^. Furthermore, the time between doses, and whether to focus on administering one dose or fully vaccinating people in each age group also varies, as can be seen from the statistics from the Nordic countries we consider in this paper. Moreover, due to severe side-effects reported, some countries have resorted to a ban of administering certain vaccine types to their citizens while other countries have chosen a path of vaccinating with a wide variety of vaccines (with a mix of the vaccines from the producers Pfizer, Moderna, Astra Zeneca and Johnson & Johnson), arguing that the effects of COVID-19 are more severe than the vaccine side-effects.

These extremes in choices related to vaccine brands offered, was especially visible in the Nordic countries, where each country chose their own path. Because of that, and due to high and rapid vaccine uptake in the Nordic countries from the get go, they are interesting to examine closer. Based on that, we examine the link between new infections and vaccination uptake through open data in the Nordic countries and ask the following two research questions: (i) how are the wave structures effected by the vaccination rate within and between various age groups in countries with high vaccination uptake? (ii) how are the key indicators of COVID-19 severity and healthcare stress level correlated? The aim is to examine the dynamic correlations between infections and vaccination rates within and between age groups in the Nordic countries. More specifically, we factor in new infections and vaccine uptake for each age group and illustrate the differences in the vaccination strategy chosen, and the effects in new cases, the vaccination rate in each age group, seriousness of illness in the population and healthcare stress level. We note that we are not clinically measuring healthcare stress level, instead we take a holistic view of healthcare stress level, thereby assessing the strain on the healthcare system via the number of hospitalisations and ICU occupancy. Furthermore, we employ the epidemic Renormalization Group (eRG) framework to illustrate wave structures and the impact of vaccinations on the wave structure and we contrast these findings with data from England^14,15^. The main contribution of this paper is the quantification of the effects of the vaccine on age groups, across countries with high vaccine uptake.

## Methods

In this section we review our methods, and the data used for this paper. Our data is extracted from open source online repositories on the virus spread through new cases reported, the seriousness of the infection through hospitalizations and deaths, the vaccinations in each country for each age group and vaccine type, and the interplay between these variables. The data analysis performed for this study is threefold: (i) frequency analysis of infections and vaccine rates by age groups; (ii) rolling correlations between vaccination strategies, severity of COVID-19 and healthcare stress level, and; (iii) we also employ the epidemic Renormalization Group (eRG) framework.

### Vaccine uptake

The vaccine uptake in the the Nordic countries was high early on, as stated earlier. In the data analyzed in this paper we look at weekly numbers of doses administered for all vaccine types allowed and administered in the Nordic countries. More specifically, we look at dosed administered of Vaxzevria—produced by AstraZeneca (AZ), Ad26.COV 2.5—produced by Janssen/Johnson & Johnson (JANSS), mRNA-1273—produced by Moderna (MOD) and Comirnaty—produced by Pfizer/BioNTech (COM) in Denmark, Finland, Iceland and Sweden until September 2021. The information for which vaccine types are used, is not available for Norway, so it is not included in the table. However, Norway has primarily been using mRNA vaccines, (see Table 1).

**Table 1:** Overview of the vaccine types used in the Nordic countries.

Country	Vaccine				Comment
	COM	MOD	AZ	JANSS	
Denmark	Yes	Yes	On hold	On hold	AZ & JANSS were only used in the beginning
Finland	Yes	Limited	Limited	No	AZ was limited to age 60+
Iceland	Yes	Yes	Limited	Yes	AZ was limited to age 55+
Norway	Yes	Yes	No	No	Decided to exclusively use mRNA vaccines
Sweden	Yes	Yes	Limited	On hold	AZ was limited to age 65+

### Data description

The goal of this research is to study the relationship between the progress of the pandemic in terms of new infections and vaccination rate per age group. As stated earlier, we focus on the Nordic countries, which have different vaccination strategies and use data up until September 2021 since that period included the largest rise in vaccination numbers. We collected data from the website of the European Center of Disease Control (ECDC). ECDC provides data that is open source and is collected through The European Surveillance System (TESSy). EU/EEA Member States are requested to report basic indicators (e.g., number of vaccine doses distributed by manufacturers, number of first and second doses administered) alongside new infections, hospitalizations, ICU occupations and deaths in their country. This data was downloaded and processed in order to conduct a retrospective analysis. For the purpose of this paper we use weekly data, divided by age group including the features (i) Number of new infections; (ii) vaccinations for the first dose; (iii) vaccinations for fully vaccinated; (iv) vaccination type for the first dose and; (v) vaccination type for fully vaccinated. Moreover, we use weekly data for the entirety of the population (not divided into age groups, since age groups were not available for this data): (vi) number of hospitalizations for the population as a whole; (vii) ICU occupations and; (viii) deaths for the population as a whole. The age groups for new infections and vaccinations do not match, as seen in Table 2.

The data utilized for the first part of the analysis is from the date when the vaccination campaign started in each country (end of December 2020) whereas the data used for the eRG part of the analysis is for the entirety of COVID-19 infections in each of the countries (since the first case was reported for each of the countries in this study). In both cases, the data is collected until September 2021. This time period was chosen as it was sufficient time to study the correlation between infections and vaccine uptake in the Nordic countries, which all started vaccinating their population rapidly as soon as they vaccines were available. Having including a longer period would not have given much additional insights for the purpose of our study, as the vaccination ratio was already quite high.

**Table 2:** The age groups for new infections and vaccinations datasets.

Data set	Age groups						
New infections	$<15$yr	$15{-}24$yr	$25{-}49$yr	$50{-}64$yr	$65{-}79$yr	80+ yr	
Vaccinations	$<18$yr	$18{-}24$yr	$25{-}49$yr	$50{-}59$yr	$60{-}69$yr	$70{-}79$yr	80+ yr

### Rolling correlations

The first and second part of the analysis illustrated in this paper is based on frequency analysis of infections and vaccine rates by age groups on the one hand, and on rolling correlations between vaccination strategies, severity of COVID-19 and healthcare stress level on the other hand. The observation period is from the last week of 2020 until week 35 in 2021.

Rolling correlations show the correlation between two time series, through a rolling window^16,17^. One of the documented benefits of such an approach is to visualize the correlation change over time^18^. Due to the fact that the pandemic has been ongoing for a while with significant shifts in the wave structure as the number of new cases increase and decrease, the rolling correlation facilitates the inspection of shifts in trends and signals of events that have occurred causing two correlated time series to deviate from each other. Uptake of vaccines could be such an event. More specifically, if there is a relationship to be found, the rolling correlation allows us to model the changes in that relationship over time, through correlations. Therefore, understanding rolling correlations for this type of data over time is both insightful and beneficial. Because the relationships between the variables included in this paper have changed rapidly, and more importantly, have changed over time, we use rolling correlations to show snapshots of those changes, see “Rolling correlation” section.

For our analysis, we calculate pairwise correlations between the various weekly time series. Due to the non-static nature of the correlation over time, we consider rolling correlations and calculate the correlation between two time series with 4 weeks of data, and a moving window of 1 week. This allows us to detect events and shifts in trends, as the pandemic evolves and vaccine update increases. For this we use the Pearson correlation coefficient^19^.

We visualize the rolling correlations using networks, where the variables are the nodes and the correlations are the edges. A positive correlation is denoted with a blue edge and a negative one with a red edge. Furthermore, the edge widths show the magnitude of the correlations, a thicker edge means a higher absolute value. For the sake of readability, we remove all edges that have an absolute value below 0.6. In addition to that, we only look at correlations between the new infections per age group and whole population indicators on the one hand and vaccine numbers per age group and whole population indicators on the other hand. Whole population indicators are weekly values for the total number of new infections (‘New Infections’), total number of people who have received the first dose (‘Total first dose’), the sum of the number of COVID-19 patients in hospital on a given day per week (‘Hospital Occupancy’), the sum of the number of COVID-19 patients in ICU on a given day per week (‘ICU Occupancy’), and the weekly count of deaths due to COVID-19 (‘Deaths’).

Finally, note that the data for Iceland and the subsequent analysis of the data shows significantly different behaviour in some of the analysis due to limited data points (the population size is 340k and when the new infection rate is low, there is low significance in the analysis due to scarcity in the data).

### Mathematical modelling

The second part of the analysis illustrated in this paper is based on the employment of the *epidemic Renormalization Group* (eRG) framework, recently developed in^20,21^. It can be mapped^21,22^ into a time-dependent compartmental model of the SIR type^23^. The eRG framework provides a single first order differential equation, apt to describing the time-evolution of the cumulative number of infected cases in an isolated region^20^. It has been extended in^14,21^ to include interactions among multiple regions of the world. The main advantage over SIR models is its simplicity, and the fact that it relies on symmetries of the system instead of a detailed description. As a result, no computer simulation is needed in order to understand the time-evolution of the epidemic even at large scales^14,21^. Recently, the framework has been extended to include the multi-wave pattern^15,24^ observed in the COVID-19 and other pandemics^25^ and even more recently to include vaccination impact^5,14^.

The Renormalization Group approach^26,27^ has a long history in physics with impact from particle to condensed matter physics and beyond. Its application to epidemic dynamics is complementary to other approaches^28–38^.

## Results

### New infections

**Figure 1:**
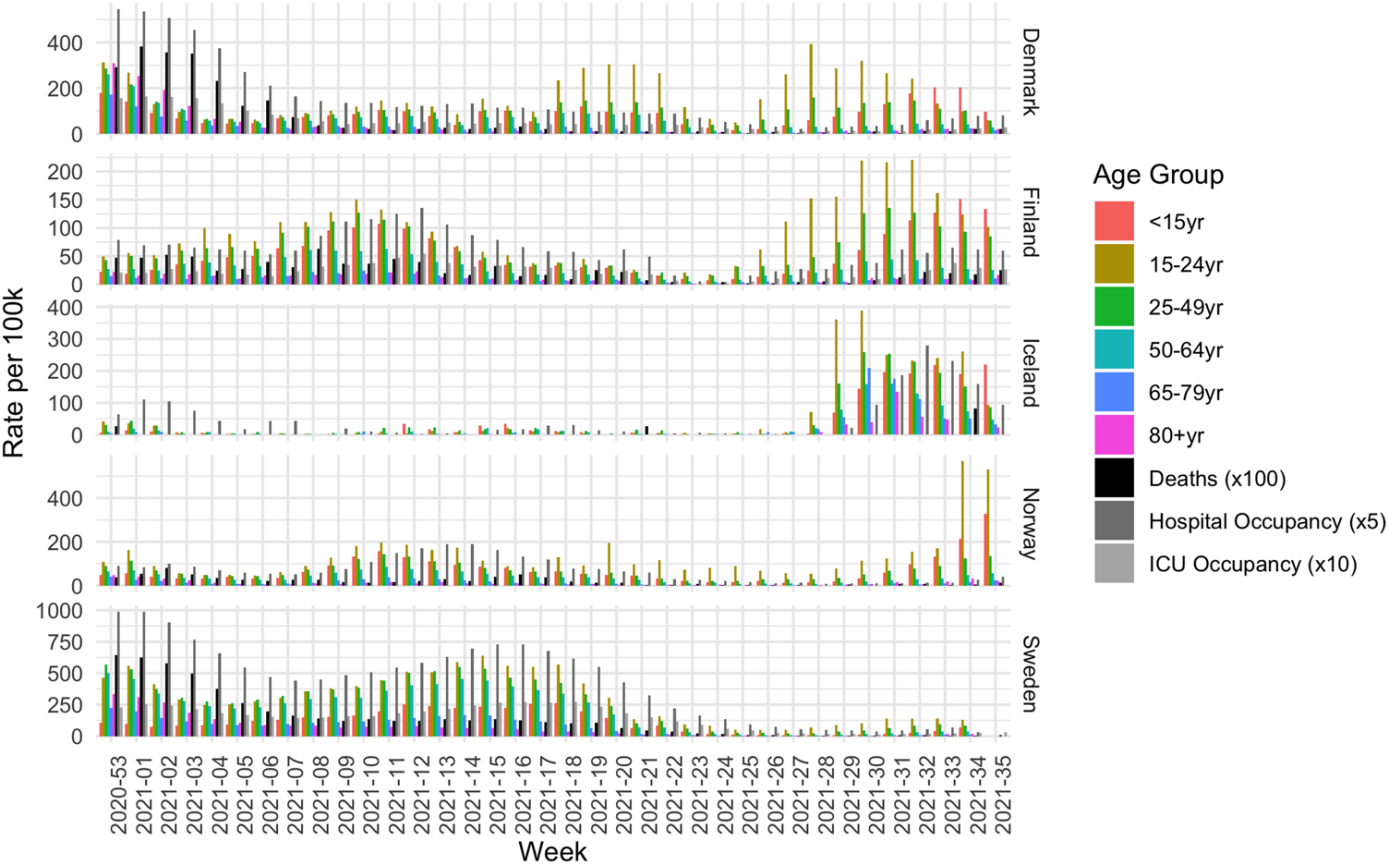
Number of new infections per 100 thousand for each age group, deaths per 10 million, hospital occupancy per 500 thousand and ICU occupancy per million for the five Nordic countries from the end of December 2020 until September 2021.

The weekly infection and hospitalization data allows us to view the progression of the pandemic. Figure 1 shows the weekly numbers of new infections per age group (normalized w.r.t. per 100,000 of the age groups’ population) during the observation period, as well as the weekly number of deaths (shown ×100 in the analysis), ICU occupancy (shown ×10 in the analysis) and hospital occupancy (shown x5 in the analysis).

In this figure we can see the waves of the pandemic for the various age groups in the five Nordic countries. In Denmark, Iceland and Sweden, the wave is going down at the start of the observation period and in all countries new waves appear, lead by the younger age groups. This is clear from the high values of the yellow and green bars, which represent people aged 15–24 and 25–49, respectively. However, this effect is less prominent in Sweden. What is also clear from these figures is that the number of hospitalizations and deaths are almost non-existent at the end of the observation period, despite the drastic increase in the number of new infections. This is a direct consequence of the high vaccination uptake. We note that this trend is less in Finland, hospitalisations and deaths are still substantial at the end of the period.

**Figure 2:**
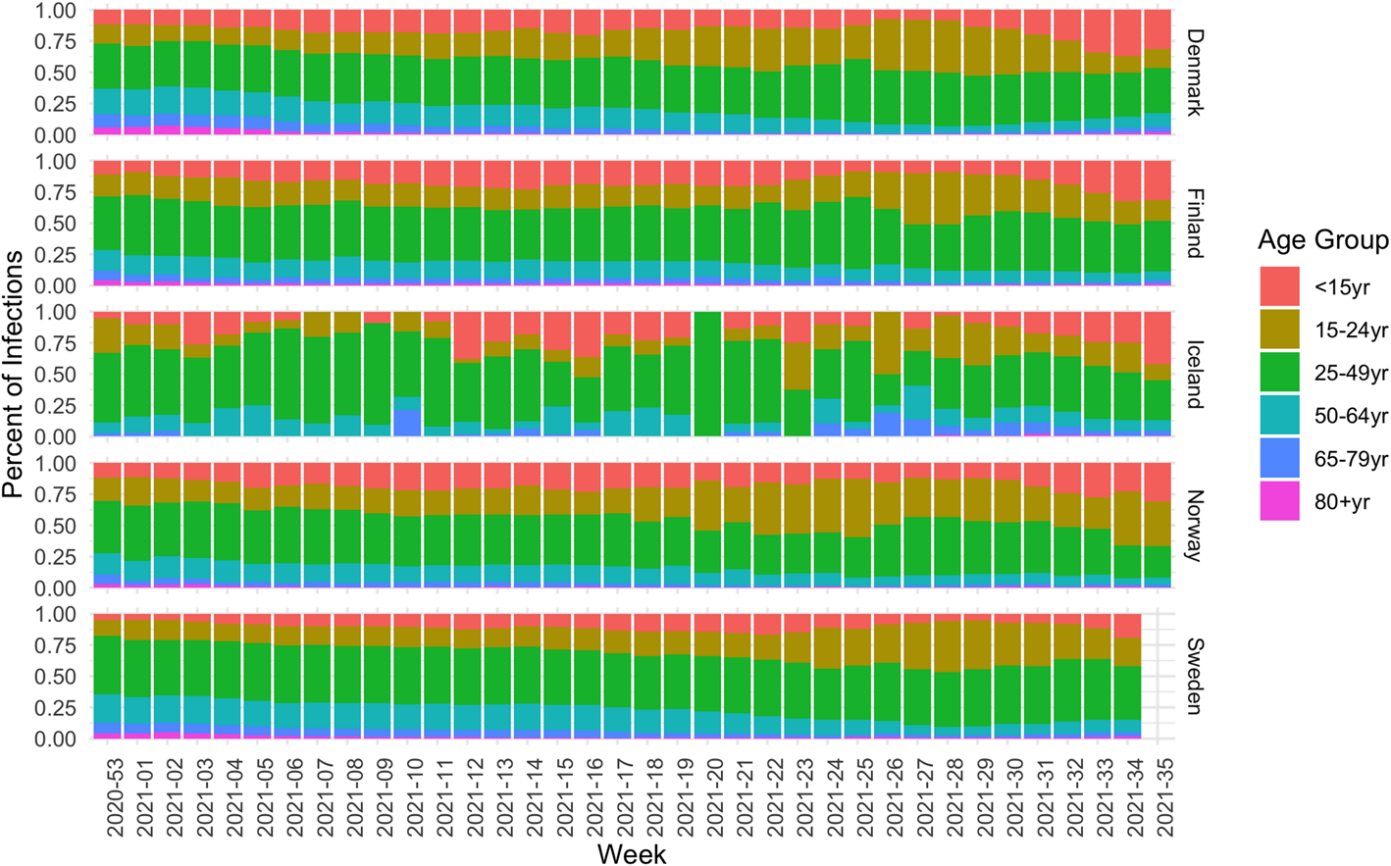
Percentage of each age group of all new infections per week in the five countries.

Figure 2 shows the ratio for each age group of the total number of new infection per week. The age group 25–49 usually makes up the largest portion, while we can see some changes in the other age groups. For example, the portion of aged 15–24 year increases over time, whereas the portion of the older generations, aged 65 and older instead diminishes. The impact of the high vaccination uptake in all five countries among the older generations is therefore clear.

### Vaccine uptake

**Figure 3:**
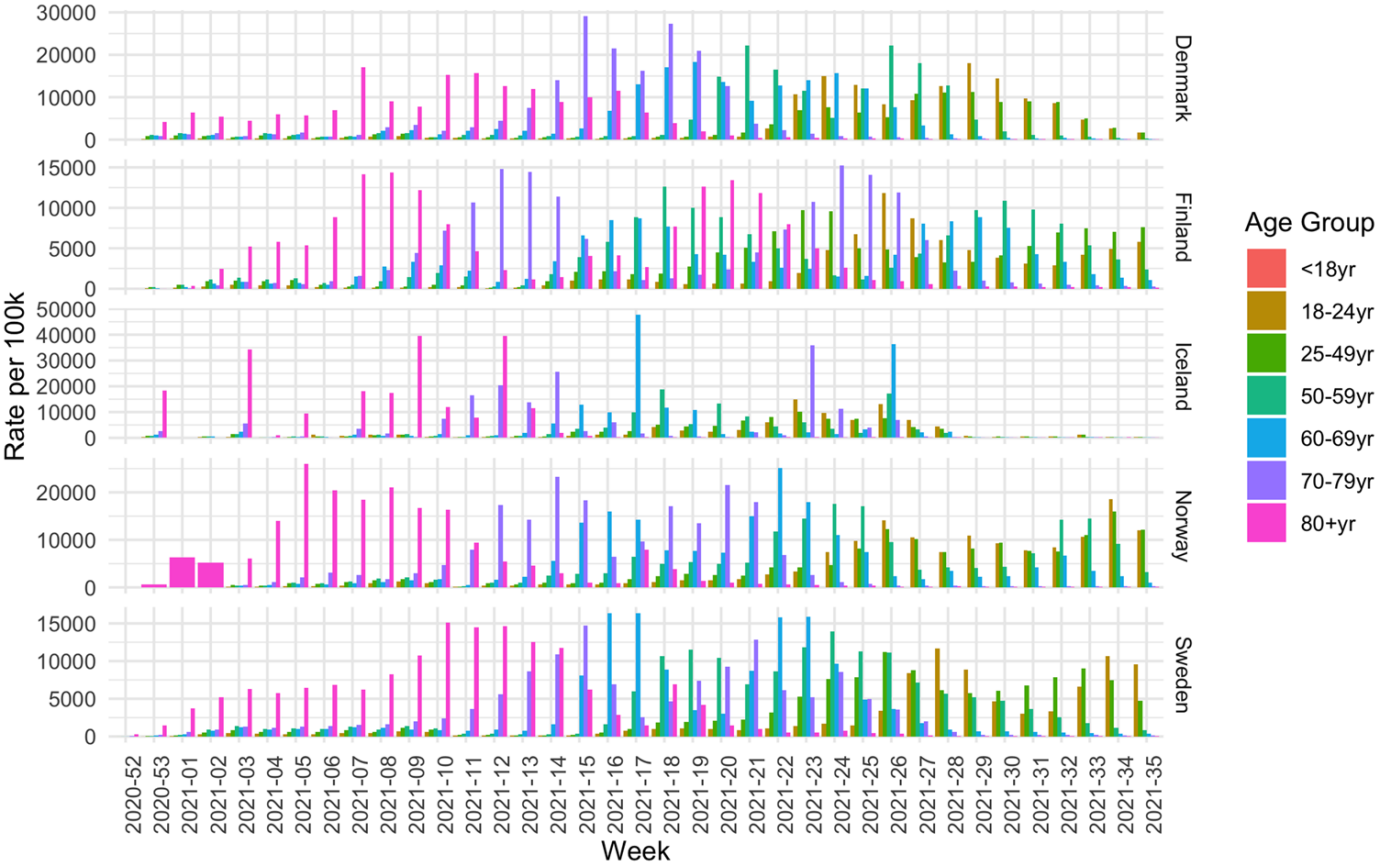
Number of vaccine doses per 100 thousand per age group given each week.

Next, we analyze the vaccination uptake in the five Nordic countries. Figure 3 shows the number of vaccines administered per week, divided by age group. Note that these are the numbers for both the first and the second dose. For each age group, the distribution is roughly bi-modal, which corresponds to the first and second dose of the vaccine. All countries show a similar trend. First the oldest generation (Age 80+, shown in pink) is vaccinated, followed by people in the other age groups. However, the time delay until younger groups are vaccinated varies between countries. For example, in Finland the 70–79 age group peaks in week 12, whereas in Denmark and Norway this peak happens in week 15. In Denmark, the 18–24 age group is vaccinated in week 22, whereas in Norway there is a delay, and this same age group is vaccinated in week 26.

**Figure 4:**
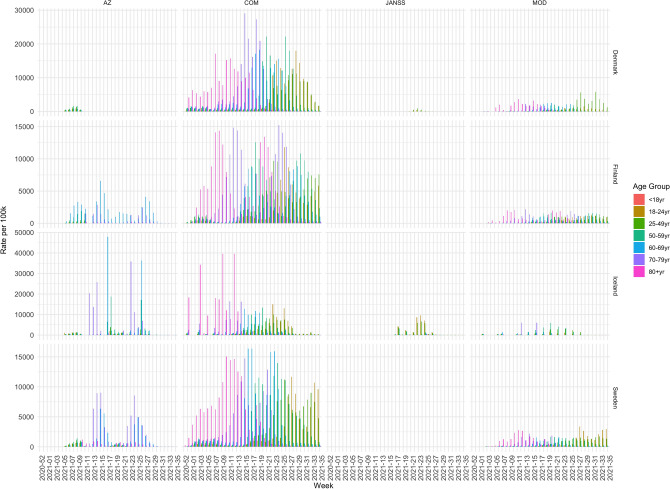
Number of vaccine doses per 100 thousand per age group and vaccine type for each week.

We take a closer look at the vaccination uptake, by looking at the trends for various vaccine types, see Fig. 4. To do that we use data which outlines weekly number of doses administered of different vaccine types (AZ, JANSS, COM and MOD) in Denmark, Finland, Iceland and Sweden. This information is not available for Norway, so it is not included in the figure. The numbers in this figure are shown per 100 thousand. Across age groups and countries the figure shows that COM is the most commonly used vaccine and that it has been given to people of all ages. The values for COM in Iceland are lower in the second half of the observation period, as the use of AZ and JANSS was quite high, compared to the other countries. AZ was given in Denmark in the first weeks, but they stopped^39^. Furthermore, we see that AZ was not given to the younger age groups in Finland, Iceland nor Sweden. JANSS was given only to a small part of the population in Denmark, and to a larger extent in Iceland, especially to the younger age groups.

**Figure 5:**
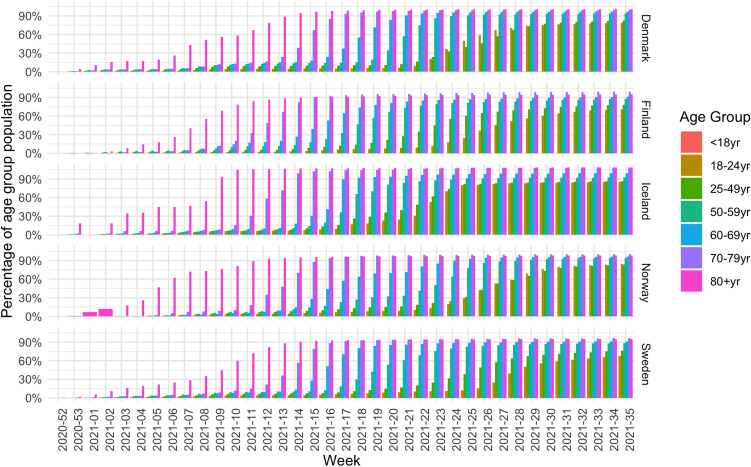
Fraction of population of each age group that has received the first vaccine dose.

**Figure 6:**
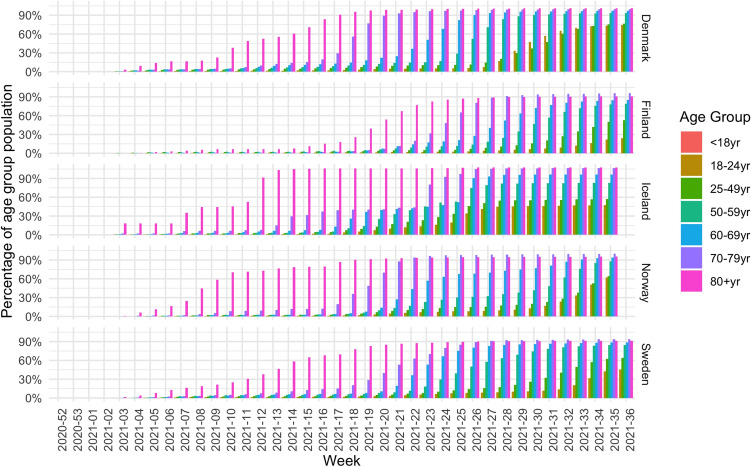
Fraction of population of each age group that has received the second vaccine dose or is fully vaccinated.

The last two figures here show the various vaccination strategies in terms of the roll out for the age groups. The effect of the strategies can be seen in Figs. 5 and 6 which show the cumulative percentage uptake for each age group of the first and the second dose respectively.

Although the countries started vaccinating their population around the same time, we can see different trends. All countries focused on the oldest generations in the first few weeks of vaccinations, with some doses given to younger people, which can most likely be explained by healthcare and front-line workers. This is however different in Norway, where only the oldest generation was included in the first 3 weeks and no significant portion of healthcare workers is detected. Furthermore, Norway also differs as the younger age groups received the vaccination with more delay compared to the other counties. By week 20, Denmark had reached 75% vaccination uptake for the 60+ age group, at week 19 in Finland, week 17 in Iceland, week 22 in Norway and week 18 in Sweden. By the end of the observation period, this goal had not been reached for the $18{-}24$ age group in Finland and Sweden and for the $25{-}49$ age group in Sweden. It was reached in week 33 in Denmark, week 25 in Iceland and week 31 in Norway.

When analyzing the data for the second dose, there is an even greater difference to be observed. Finland lags behind the other countries when it comes to starting the administration of the second dose. For the older generations (60+), Denmark, Iceland and Sweden had reach 75% vaccination uptake for that population in weeks 25 and week 26, whereas Finland and Norway were weeks behind, reaching this milestone in week 31. By the end of the observation period, Finland, Norway and Sweden are clearly lagging behind in delivering the second dose, especially for people under 50 years old.

**Table 3:** The week number in 2021 where at least 10% of the population within the respective age groups had received the first vaccine dose (1st) and were fully vaccinated (full).

Age group	18–24		25–49		50–59		60–69		70–79		80+	
Uptake country	1st	Full	1st	Full	1st	Full	1st	Full	1st	Full	1st	Full
Denmark	W23	W28	W16	W22	W9	W19	W9	W14	W9	W12	W1	W5
Finland	W23	W33	W16	W28	W11	W23	W9	W21	W9	W21	W4	W15
Iceland	W17	W19	W15	W18	W14	W18	W13	W17	W10	W13	W0	W3
Norway	W23	W30	W18	W25	W14	W22	W14	W20	W10	W14	W1	W5
Sweden	W22	W30	W18	W24	W13	W22	W13	W20	W10	W14	W2	W6
EU/EEA	W20	W25	W16	W22	W12	W19	W12	W18	W10	W14	W4	W8

Now we can compare the new infection rates to vaccination strategies, by looking at Table 3. This table shows the week number in which each country reached a 10% vaccination level in each age group for the first and second dose respectively. As such, the table illustrates when vaccinations were well underway in each age group and can be seen as a representation of the vaccination strategy. For example, Iceland was the first country to reach this level in the younger categories for both first dose and for fully vaccinated. To reach this goal, Iceland used all vaccine types, whereas the other countries refrained from using JANSS and AZ (see Table 1). Furthermore, Finland reached the 10% level for the first dose earlier than the other countries, but lagged behind for the second dose, indicating that Finland prioritized the administration of the first dose to a large part of the population instead of fully vaccinating a smaller portion of the population.

The impact of the vaccination strategies on new infections is clear. Finland has a larger wave at the end of the observation period, a wave which is especially visible among the younger generations. Finland also has a more severe wave, with less reduction in the number of hospitalizations and deaths, compared to the last wave and the other countries.

However, in all countries, the new wave which starts in the summer months of 2021 is dominated by the younger age groups who are getting infected to a larger extent compared to the older age groups.

### Rolling correlation

**Figure 7:**
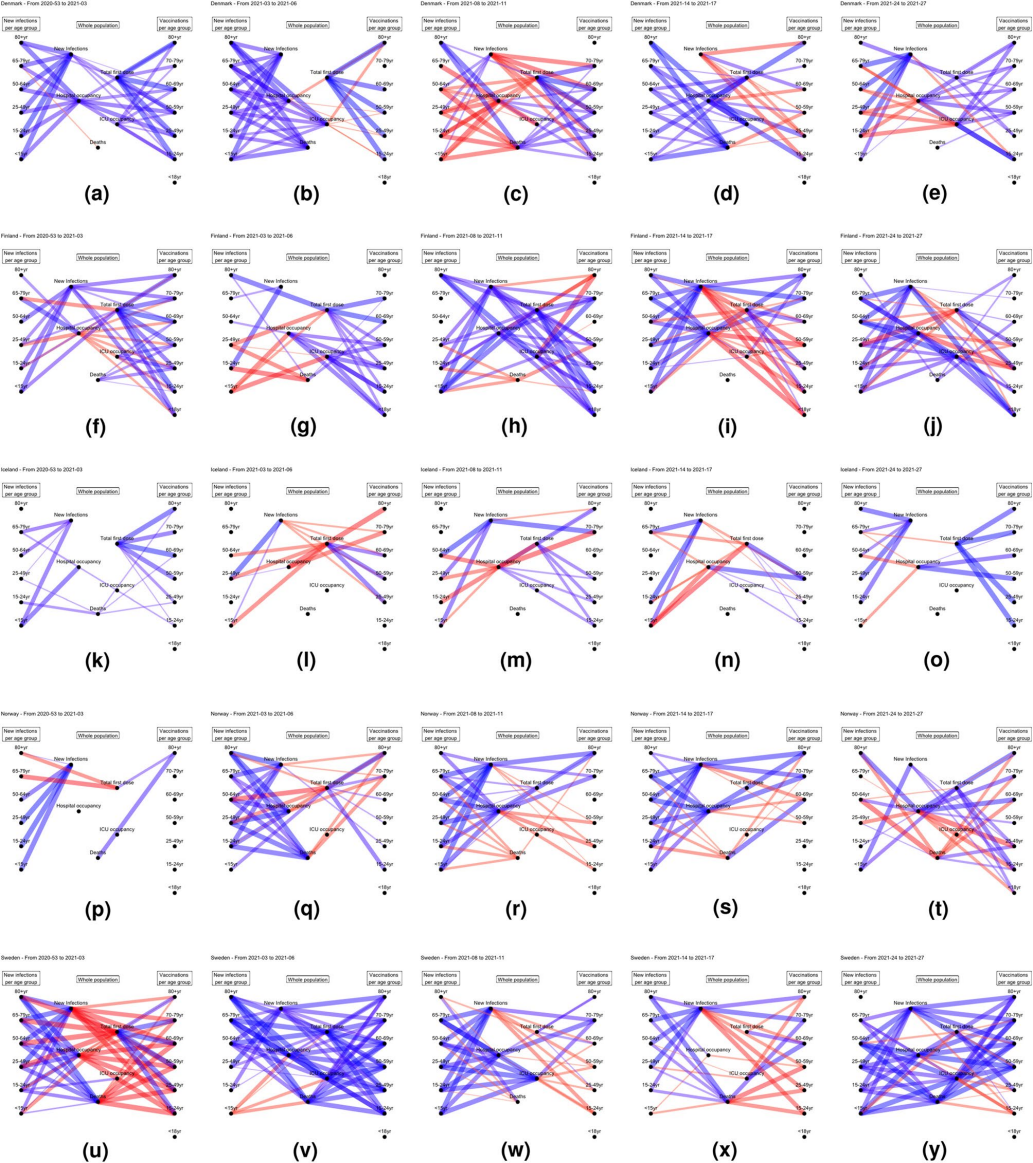
Rolling correlations between changes in population indicators (middle) and infections in age groups (left) and vaccination of age groups (right). Rows from top to bottom: Denmark, Finland, Iceland, Norway, Sweden. Column left to right: From 2020-53 to 2021-03,From 2021-03 to 2021-06, from 2021-08 to 2021-11,From 2021-14 to 2021-17,From 2021-24 to 2021-27. Blue indicates a positive correlation and red a negative correlation, and a thicker edge indicates a stronger correlation. Correlations with an absolute value below 0.6 are omitted.

Now we look at the rolling correlations to inspect trends in the relationships between variables. Figure 7 shows the rolling correlations for five periods in time, in the five Nordic countries. Each figure has three parts: population indicators (middle), infections in age groups (left) and vaccination of age groups (right). The five periods are: (i) Weeks 2020-53 to 2021-03, (ii) Weeks 2021-03 to 2021-06, (iii) Weeks 2021-08 to 2021-11, (iv) Weeks 2021-14 to 2021-17, (v) Weeks 2021-24 to 2021-27. The left side of each of these figures shows the progression of the pandemic, in terms of which age groups are getting infected and hospitalized, where as the right hand side shows the vaccination strategy.

We see interesting trends for all countries. Denmark and Sweden show similar patterns at the start of the observation period, see Fig. 7a,b for Denmark and 7u,v for Sweden. As is visible in Fig. 1, the wave is going down and so are the number of infections and hospitalizations, as indicated by the positive correlations shown here. However, in Denmark about the same number of people were vaccinated in all age groups about the same number of people per week in the beginning, while in Sweden the number grew every week during the first 4 weeks of vaccinations. This is why there is a negative correlation between the vaccinations per age group and the indicators of the whole population in Fig. 7u.

In Sweden, infections of all age groups are positively correlated with new infections in the beginning. Looking towards the seriousness of the illness, we see that all age groups are positively correlated with hospital occupancy and deaths, whereas only people aged 18–24 years are correlated with ICU occupancy. In contrast to Fig. 1, infections, deaths and hospital occupancy show a negative trend in the first 4 weeks, while the ICU occupancy is more or less stable. Moving on to weeks 3 to 6, the highest five age groups have a positive correlation with new infections; indeed the number of infections are increasing for all of these age groups.

In Finland, at the end of the observation period (Fig. 7j, all age groups except the oldest have a high positive correlation with new infection, whereas only the younger groups ($<15$, $15{-}24$, $24{-}49$ and $50{-}64$ are correlated with hospitalizations and ICU occupancy, indicating that these are the age groups with more severe illness.

Norway looks different from the other observed countries when it comes to the vaccination correlations. For each period, there are fewer positive correlations between vaccinated age groups and total vaccines administered compared to what we observe for the other countries. This might indicate that the vaccination strategy was more divided and that the focus was to finish vaccinating one age group before starting the next one.

The number of new infections in each age group is positively correlated with total number of new infections in variable ways. In these 25 figures we get a positive correlation between new infections: 12 times for the oldest age groups (80+), 17 times for the second highest ($65{-}79$) and 19 times or more for the remaining age groups. This means that the oldest generations that were vaccinated first seem to be better protected when it comes to new infections.

### eRG results

**Figure 8:**
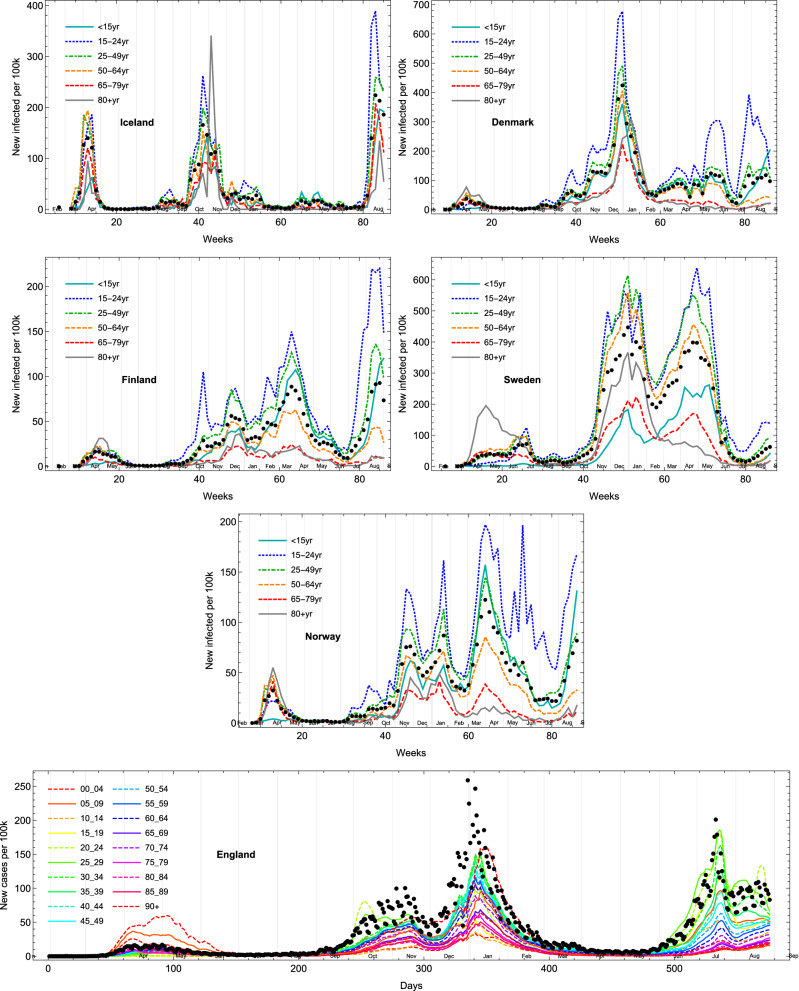
Number of cases per 100k inhabitants from each age class within Iceland, Denmark, Finland, Sweden, Norway and England. Black dots represents the number of new cases per 100k inhabitants for all age groups.

Now we would like to study the evolution of the epidemic waves for each age group in the Nordic countries through the scope of the epidemic Renormalisation Group (eRG) formalism. The eRG fit parameters can be seen in Table S1. First, when examining the data for the number of new cases per 100k inhabitants from each age group (see Fig. 8), the 80+ category behaves differently, across the different waves. We can see that the 80+ age group has fewer cases for the later waves and that trend is increasingly visible after the beginning of the vaccination campaigns early 2021. Furthermore, we observe that the 15–24 age group represents the major part of the new cases reported while being one of the least vaccinated group.

One way to quantify these observations is to study the data through the eRG formalism^20^ and the logistic function:

1 $$\begin{aligned} I(t) = \frac{ae^{\gamma (t-t_0)}}{1 + e^{\gamma (t-t_0)}} \end{aligned}$$

**Figure 9:**
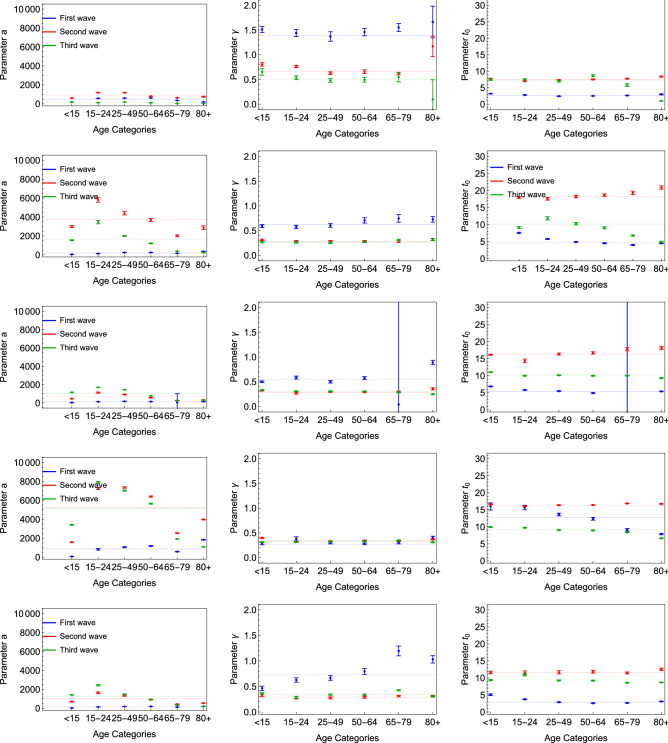
Fit parameters for Iceland, Denmark, Finland, Sweden and Norway.

Each age group, wave or country is then comparable with another for both the total number of cases (per 100k inhabitant) per wave “*a*” and the infection rate $\gamma$ as can be seen in Fig. 9. In this part of the analysis, we also take into account a time shifting parameter $t_0$ representing the relative timing of the peak among the age groups for each wave. First, we can see that the parameter *a* is always smaller for the first wave compared to the next ones. This observation can be explained by the testing capacity difference between the beginning of the COVID-19 outbreak across the world, compared to later times. A lower number of tests taken logically brings a lower number of reported cases and less relevance of the normalisation of the number of cases. This may also explain the over-representation of the 80+ age group during the first wave, as retirement homes were tested to a larger extent in some of the observed countries. However, the comparison between the parameter *a* within each wave shows that the parameter is smaller for higher age groups for the third wave. This may be a direct effect and reflection of the vaccination strategy to vaccinate mostly the highest age groups first.

To further validate the action of the vaccination strategies on the evolution of the pandemic, we also examined any correlation between the vaccination percentage and the eRG parameters. The eRG parameters integrate the time evolution of the whole wave while the vaccination rates have been changing over time. To take this into account, therefore, we show in Figs. 11 and 12 the rolling correlation between the time series of the vaccination percentage per week, since the beginning of the campaign and the fixed eRG parameters for the relevant wave. From our previous works^14^, we know that only the vaccination before the peak has a measurable impact on the wave. As a sanity check, we report the correlation with the eRG parameters of previous waves (two in the Nordic countries and three in England) where no meaningful correlation should be observed. If any correlation exists, one could expect that it would be seen only for the last wave, which takes place after the vaccination campaigns begun, and close to the peak of the wave. We expect this correlation to be close to minus one, as more vaccinated individuals bring less infections, so a smaller *a* and a smaller $\gamma$, while correlation for other waves should be random. The results shown in Fig. 12 are, therefore, coherent for parameters *a* and $\gamma$ and for almost any country. However, a problem seems to appear for $\gamma$ in Denmark, but when looking at the behavior of the third wave in Denmark from Fig. 8, one can see that there was hardly any rise in the wave for older age groups; we therefore conclude that the fit is sub-optimal here.

**Figure 10:**
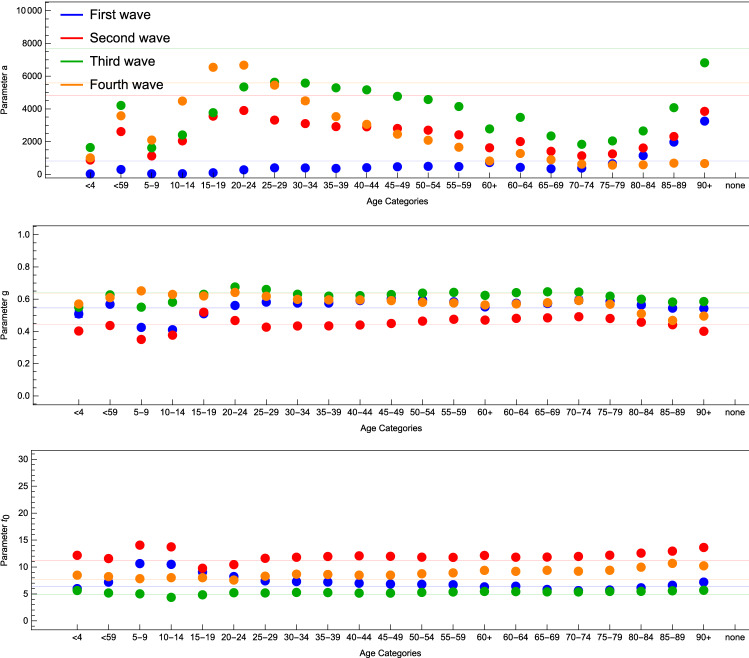
Fit parameters for England.

We would like to compare our previously outlined results for the Nordic countries to England. We chose England because much like the Nordic countries which we have focused on, England had high vaccination uptake early. Observing the fit parameters for England, the vaccination campaign started early and the fourth wave took place 7–8 months after the vaccination started when compared to the start of the vaccination. The population was highly vaccinated in April 2021 (the plateau of vaccinations was reached for the 70+ age group then). In Fig. 10, we can see the fit parameters for the different age groups and waves in England. What can be observed here is that the distribution for *a* changes significantly for the fourth wave, where older age groups have a smaller parameter, while the age groups for 10–24 years old seem to have an high number of new infections. The shrink for older age groups can be explained by the high number of vaccinations administered, while the rise for younger age groups can be explained by the delta variant that was the primary variant and source for infections during the fourth wave in combination with comparatively lower vaccination uptake for those age groups.

**Figure 11:**
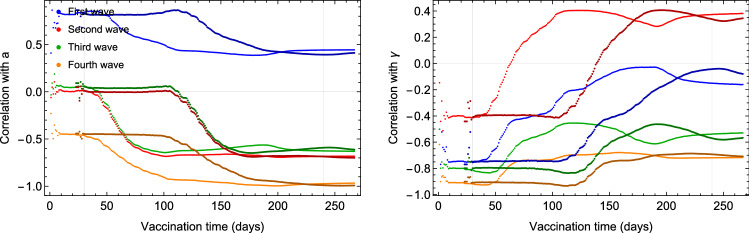
Correlation between vaccination and eRG parameters for the last wave in England. The vertical grey lines indicate the peak timing of the two last waves in England.

We would now like to turn the attention towards the correlation between vaccination percentage and eRG parameters among the different age groups (see Fig. 11). Here we observe that the fourth wave eRG parameters have a clear anti-correlation with the vaccination percentage, and similarly for $\gamma$.

The results further corroborate the findings in our previous work^14^ about the US vaccination campaign impact on the epidemiological data. Indeed, the most vaccinated age groups have a significant reduction in *a* and $\gamma$.

**Figure 12:**
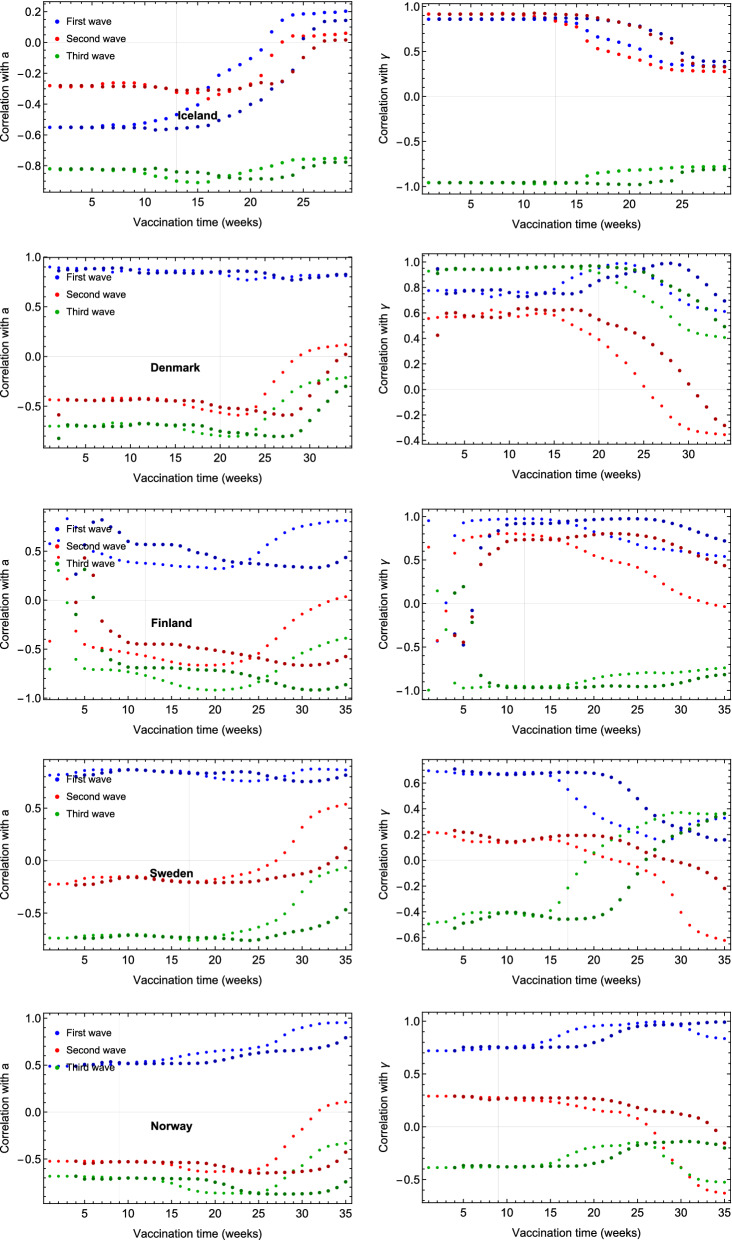
Correlation between the last wave fit parameters and vaccination percentage for Iceland, Denmark, Finland, Sweden and Norway. The vertical grey line indicates the peak timing of the last wave in the country.

## Discussion

In this paper we examine the deployment of vaccines in the Nordic countries in a comparative analysis. Our main results are the quantification of the impact of the vaccination campaigns on age groups through epidemiological data, across countries with high vaccine uptake. Through our findings, we clearly show that the vaccinations have clearly hindered the spread of the virus.

The full extent of the healthcare stress level is difficult to predict until years have passed since the pandemic fully subsides^40^. Up until now, there have been attempts that focus on assessing the impact on a micro level whereas we, in this paper, analyze the impact on a meso and macro level instead; through data. Thereby, we analyze the severity and healthcare stress levels utilizing data on new infections, hospitalizations, intensive care unit (ICU) occupancy and deaths. As stated earlier, the healthcare stress level is not measured by utilizing clinical stress level instruments, but instead we rely heavily on a data-driven approach. What can be derived from the results is that healthcare stress level has been high, i.e., that most countries have been operating at capacity when it comes to hospital resources at different time periods during the COVID-19 pandemic.

For that purpose, we consider key indicators of the severity of the COVID-19 disease and the stress level in healthcare in terms of the number of hospitalizations, ICU occupancy and death. Although hospitalizations and ICU occupancy relate directly to the healthcare stress level, all three can be seen as key indicators for the severity. Research has shown that vaccinations reduce the severity of the illness, which we clearly observe through our analysis too. Table 4 shows the number of hospital and ICU beds, as well as the number of physicians and nurses in the five Nordic countries. According to these numbers, the healthcare system in Norway and Finland seem best positioned to handle a major health crisis such as a pandemic, although Finland has the lowest number of physicians. In countries with few hospital and ICU beds, it is even more important to reduce the stress level of the healthcare system.

**Table 4:** The number of hospital and ICU beds, doctors and nurses in the five Nordic countries. Source: https://tradingeconomics.com/.

Country	Hospital beds (per 1000)	ICU beds (per 100k)	Medical doctors (per 1000)	Nurses (per 1000)
Denmark	2.57	245.88	4.27	11.24
Finland	3.35	307.55	3.33	15.73
Iceland	2.91	263.29	3.94	14.85
Norway	3.60	342.52	5.53	20.83
Sweden	2.22	234.50	4.32	11.49

Coupled with that analysis, we also analyze the impact of the various vaccine types, vaccination rate on the spread of the virus in each age group for Denmark, Finland, Iceland, Norway and Sweden from the start of the vaccination period in December 2020 until September 2021. Our findings from the analysis clearly shows that vaccines markedly reduce the number of new cases and the risk of serious illness.

What can be clearly derived from our results is that the vaccination strategies vary to a large extent. The analyzed countries all led focused vaccination campaigns with high vaccination uptake early. However, there is a clear difference for Norway, which decided to select mRNA instead of a wide variety of vaccines for their citizens which resulted in a slower vaccination uptake early on, compared to the other studied countries. Moreover, we see that Sweden had a steadier death rate and did therefore not have a similar decline in death rate early on compared to what could be observed for the other studied countries. This may be a consequence of a different strategy that Sweden chose to battle the outbreak of the virus early on^41^. In the Nordic countries, as well as in England, the oldest generations seem to be better protected against COVID-19 as seen by the low numbers of infections and less correlation with the total number of new infections. This can probably be attributed to the high ratio of fully vaccinated people among these cohorts. From the data, we see that Norway and Finland had different strategies from the other countries. Norway focused more on completing the vaccinations for one age group before moving on to a younger one, whereas Finland prioritized the administration of the first dose to as many as possible. The impact of these decisions is clear in the data. At the end of our analysis period, the infection wave was growing fast for the $<15$ and $15{-}24$ age groups, subsiding only after our analysis period ended. In Finland however, the situation at the end of the analysis period was graver. Not only were the infections numbers high and rising for $15{-}49$ year old people, but the number of hospitalizations, ICU occupancy and deaths, had not decreased at the same rate as in the other countries. Regarding the use of different vaccination types we show that the use of AstraZeneka was discontinued for most countries early. We also observe that Johnson & Jonson/Jansen was primarily administered in Iceland but not used to the same extent in the other Nordic countries; Iceland later resulted in a booster dose for everyone that had accepted that particular vaccine type. Moreover, we show that Pfizer was used to most of all vaccine types in the data-set while Moderna had a steady spread across all countries.

These results combined show that the wave structures and new infections is highly effected by the vaccination rate within and between various age groups in countries with high vaccination uptake. We also show that when the vaccination rate in a specific age group has reached a plateau, then the new infection rate follows and decreases drastically for that age group. Furthermore, we clearly show a wave on the rise for the youngest and least vaccinated part of the population at the end of the study period.

Regarding the data used, it is concerning that the data differs, depending on the country. For instance, there is no information on deaths divided into age categories for European countries whereas that data is readily available for England. Also, Norway did not report all data (ICU occupancy and vaccination type is missing), which has implications for all retrospective data analysis. The age groups in England are also significantly different compared to the ones used in the Nordic countries, making a comparison cross Europe as a whole difficult to achieve. Having unified data standards would make the work in cleaning the data, less time consuming.

To sum up, we have examined the impact of vaccine uptake on the pandemic dynamics in the Nordic countries. In these countries, the vaccination campaigns has been notably efficient with a significantly high fraction of the population already vaccinated. Our analyses clearly demonstrate that a successful vaccination strategy is paramount to reduce stress on general health and its healthcare system. The results suggest that for countries where the fraction of unvaccinated people is still high, measures are required to either increase the vaccination rate or to continue with non-pharmaceutical measures such as social distancing.

Whether the resistance to vaccinations can be attributed to cultural factors such as obedience to authority and regulatory compliance remains an interesting open question. Early work on social order and disorder when it comes to non-pharmaceutical measures during COVID-19 has been published^42^ whereas obedience to authority in relation to vaccinations has not been studied.

## Supplementary Information

Supplementary Table S1.
