# Supplementary material for: The quantification of vaccine uptake in the Nordic countries and impact on key indicators of COVID-19 severity and healthcare stress level via age range comparative analysis
Source: Sci Rep. 2022 Oct 7;12:16891. doi: 10.1038/s41598-022-21055-0 (PMC9542476; doi:10.1038/s41598-022-21055-0)
Supplement: Supplementary file 1 — Supplementary Table S1. [file 41598_2022_21055_MOESM1_ESM.pdf]

Additional information

eRG parameters tables

Table S1. eRG fit parameters.

Country	Wave #	age group	a	γ	t_0	% 1st dose	% 2nd dose
Iceland	1	<15	171(2)	1.51(7)	3.2(4)	0	0
		15-24	573(5)	1.44(8)	2.79(4)	0	0
		25-49	596(7)	1.37(10)	2.43(6)	0	0
		50-64	616(6)	1.46(8)	2.51(4)	0	0
		65-79	346(3)	1.55(8)	2.65(4)	0	0
		80+	188(6)	1.7(4)	3.0(2)	0	0
		50-79	513(5)	1.48(8)	2.54(4)	0	0
	2	<15	601(8)	0.80(4)	7.7(7)	0	0
		15-24	1183(12)	0.76(3)	6.97(6)	0	0
		25-49	1172(20)	0.63(3)	7.33(10)	0	0
		50-64	798(17)	0.66(4)	7.5(2)	0	0
		65-79	628(11)	0.62(3)	7.8(2)	0	0
		80+	750(33)	1.2(3)	8.4(2)	0	0
		50-79	733(14)	0.64(4)	7.6(2)	0	0
	3	<15	169(7)	0.65(7)	7.4(3)	(18-24) 6.7	0.7
		15-24	108(4)	0.54(4)	7.5(2)	(25-49) 7.1	1.9
		25-49	189(7)	0.48(4)	6.9(3)	(50-59) 9.1	2.3
		50-64	106(7)	0.49(5)	8.6(4)	(60-69) 18.2	5.1
		65-79	32(2)	0.6(1)	5.8(4)	(70-79) 90.7	32.9
		80+	0	0	0	(80+) 98.5	96.9
		50-79	77(4)	0.48(5)	8.2(3)	30.3	10.0
Denmark	1	<15	1546(43)	0.27(2)	9.1(4)	0	0
		15-24	3454(170)	0.26(2)	11.8(5)	0	0
		25-49	1991(65)	0.25(2)	10.2(4)	0	0
		50-64	1218(35)	0.27(2)	9.1(4)	0	0
		65-79	392(8)	0.31(2)	6.8(3)	0	0
		80+	225(4)	0.32(3)	4.9(3)	0	0
		50-79	211(5)	0.713(6)	4.3(2)	0	0
	2	<15	2989(99)	0.31(2)	17.9(3)	0	0
		15-24	5794(236)	0.28(2)	17.6(4)	0	0
		25-49	4394(173)	0.29(2)	18.2(4)	0	0
		50-64	3686(148)	0.29(2)	18.6(4)	0	0
		65-79	2017(108)	0.28(2)	19.3(5)	0	0
		80+	2874(189)	0.32(3)	20.9(5)	0	0
		50-79	2957(128)	0.28(2)	18.8(4)	0	0
	3	<15	1546(44)	0.27(2)	9.1(4)	(18-24) 5.5	3.2
		15-24	3454(170)	0.26(2)	11.8(5)	(25-49) 10.6	7.0
		25-49	1991(65)	0.25(2)	10.2(4)	(50-59) 19.3	10.8
		50-64	1218(35)	0.27(2)	9.1(4)	(60-69) 71.9	18.1
		65-79	392(8)	0.31(2)	6.8(3)	(70-79) 97.1	76.8
		80+	225(4)	0.32(3)	4.9(3)	(80+) 99.6	97.6
		50-79	857(23)	0.28(2)	8.6(3)	58.1	99.6
Finland	1	<15	39.4(6)	0.50(2)	6.84(10)	0	0
		15-24	130(3)	0.58(4)	5.8(2)	0	0
		25-49	174(3)	0.50(3)	5.5(2)	0	0
		50-64	148(3)	0.57(3)	4.9(1)	0	0
		65-79	0	0	0	0	0

Continued on next page

Table S1– continued from previous page

Country	Wave #	age group	a	γ	t_0	% 1st dose	% 2nd dose
		80+	152(2)	0.89(5)	5.39(7)	0	0
		50-79	106(2)	0.59(4)	4.8(2)	0	0
	2	<15	457(9)	0.321(7)	16.1(2)	0	0
		15-24	1128(63)	0.27(2)	14.3(6)	0	0
		25-49	920(34)	0.29(2)	16.3(3)	0	0
		50-64	572(25)	0.29(2)	16.7(4)	0	0
		65-79	257(17)	0.30(2)	18.8(5)	0	0
		80+	324(26)	0.36(3)	18.1(6)	0	0
		50-79	428(21)	0.29(2)	17.0(4)	0	0
	3	<15	1161(9)	0.334(7)	11.07(8)	(18-24) 2.1	0.6
		15-24	1704(14)	0.308(8)	9.97(10)	(25-49) 4.9	2.0
		25-49	1441(11)	0.308(7)	10.14(10)	(50-59) 9.5	2.3
		50-64	771(6)	0.305(7)	9.96(9)	(60-69) 14.3	20.2
		65-79	282(3)	0.285(7)	10.0(2)	(70-79) 20.2	0.8
		80+	310(4)	0.248(8)	9.3(2)	(80+) 78.3	6.5
		50-79	547(4)	0.301(7)	9.97(9)	14.2	1.5
Sweden	1	<15	78(11)	0.29(4)	16(2)	0	0
		15-24	825(87)	0.38(4)	15.7(7)	0	0
		25-49	1056(50)	0.29(2)	13.6(5)	0	0
		50-64	1179(50)	0.28(2)	12.3(4)	0	0
		65-79	576(21)	0.30(3)	9.1(4)	0	0
		80+	1837(37)	0.40(3)	7.9(2)	0	0
		50-79	910(39)	0.27(2)	11.4(5)	0	0
	2	<15	1580(25)	0.40(2)	16.6(2)	0	0
		15-24	7263(111)	0.318(9)	16.0(2)	0	0
		25-49	7365(77)	0.334(7)	16.3(1)	0	0
		50-64	6404(66)	0.345(7)	16.40(9)	0	0
		65-79	2540(30)	0.339(7)	16.9(2)	0	0
		80+	2975(42)	0.365(8)	16.73(9)	0	0
		50-79	4663(50)	0.343(7)	16.5(1)	0	0
	3	<15	3426(41)	0.312(9)	9.9(2)	(18-24) 4.5	1.7
		15-24	7913(82)	0.314(8)	9.7(2)	(25-49) 7.1	3.1
		25-49	7040(61)	0.322(8)	9.1(1)	(50-59) 10.5	5.2
		50-64	5658(53)	0.328(9)	8.9(1)	(60-69) 27.9	6.1
		65-79	1916(15)	0.350(9)	8.36(9)	(70-79) 79.0	12.8
		80+	1084(11)	0.31(2)	6.6(2)	(80+) 92.1	65.0
		50-79	3970(35)	0.333(9)	8.8(1)	33.4	7.0
Norway	1	<15	32(2)	0.46(5)	5.1(3)	0	0
		15-24	137(3)	0.63(5)	3.7(2)	0	0
		25-49	187(3)	0.67(5)	2.9(1)	0	0
		50-64	196(3)	0.79(6)	2.58(9)	0	0
		65-79	126(2)	1.2(1)	2.67(8)	0	0
		80+	205(3)	1.03(8)	3.09(8)	0	0
		50-79	166(3)	0.90(7)	2.61(9)	0	0
	2	<15	703(31)	0.33(3)	11.6(4)	0	0
		15-24	1636(94)	0.28(3)	11.5(6)	0	0
		25-49	1335(66)	0.28(3)	11.6(5)	0	0
		50-64	919(42)	0.29(2)	11.8(5)	0	0
		65-79	432(14)	0.31(2)	11.5(3)	0	0
		80+	546(22)	0.31(2)	12.5(3)	0	0
		50-79	717(30)	0.30(2)	11.7(4)	0	0
	3	<15	1415(21)	0.36(2)	9.4(2)	(18-24) 3.2	0.8

Continued on next page

Table S1– continued from previous page

Country	Wave #	age group	a	γ	t_0	% 1st dose	% 2nd dose
		15-24	2432(82)	0.29(3)	11.0(4)	(25-49) 4.6	1.7
		25-49	1478(20)	0.34(2)	9.3(2)	(50-59) 7.6	2.5
		50-64	903(10)	0.34(2)	9.2(2)	(60-69) 5.6	2.0
		65-79	311(2)	0.43(2)	8.58(8)	(70-79) 17.9	8.2
		80+	178(2)	0.308(6)	8.69(8)	(80+) 80.2	23.3
		50-79	657(7)	0.36(2)	9.0(2)	9.5	3.8
England	1	<4	28.3(4)	0.51(3)	6.0(1)	0	0
		<60	300(2)	0.57(1)	7.18(4)	0	0
		5-9	38.9(6)	0.425(7)	10.63(8)	0	0
		10-14	45.9(7)	0.410(7)	10.59(8)	0	0
		15-19	101.7(6)	0.510(5)	9.06(3)	0	0
		20-24	279(2)	0.561(8)	8.22(4)	0	0
		25-29	401(3)	0.58(1)	7.43(4)	0	0
		30-34	397(3)	0.58(1)	7.30(4)	0	0
		35-39	366(2)	0.58(1)	7.21(4)	0	0
		40-44	412(3)	0.59(1)	6.99(4)	0	0
		45-49	467(3)	0.60(2)	6.83(4)	0	0
		50-54	491(3)	0.59(1)	6.78(3)	0	0
		55-59	478(3)	0.58(2)	6.71(4)	0	0
		60+	721(6)	0.55(2)	6.31(6)	0	0
		60-64	430(3)	0.57(2)	6.43(5)	0	0
		65-69	344(3)	0.57(2)	5.83(7)	0	0
		70-74	385(4)	0.59(2)	5.61(7)	0	0
		75-79	650(6)	0.59(2)	5.73(7)	0	0
		80-84	1148(10)	0.56(2)	6.14(6)	0	0
		85-89	1973(14)	0.54(2)	6.61(5)	0	0
		90+	3255(18)	0.542(9)	7.19(4)	0	0
	2	<4	876(5)	0.402(3)	12.17(4)	0	0
		<60	2616(14)	0.436(3)	11.57(4)	0	0
		5-9	1127(16)	0.350(3)	14.06(9)	0	0
		10-14	2046(33)	0.377(5)	13.7(1)	0	0
		15-19	3558(43)	0.52(2)	9.77(8)	0	0
		20-24	3903(25)	0.468(6)	10.44(4)	0	0
		25-29	3317(14)	0.426(3)	11.62(3)	0	0
		30-34	3103(11)	0.434(2)	11.81(2)	0	0
		35-39	2920(10)	0.434(2)	11.95(2)	0	0
		40-44	2906(8)	0.440(2)	12.07(2)	0	0
		45-49	2807(7)	0.449(2)	11.98(2)	0	0
		50-54	2701(8)	0.464(2)	11.82(2)	0	0
		55-59	2418(8)	0.475(2)	11.78(2)	0	0
		60+	1625(5)	0.470(2)	12.15(2)	0	0
		60-64	2006(6)	0.481(2)	11.82(2)	0	0
		65-69	1412(5)	0.484(3)	11.84(2)	0	0
		70-74	1137(4)	0.491(2)	11.96(2)	0	0
		75-79	1251(4)	0.480(2)	12.19(2)	0	0
		80-84	1614(5)	0.457(2)	12.57(2)	0	0
		85-89	2316(8)	0.441(2)	12.95(2)	0	0
		90+	3851(29)	0.401(3)	13.63(5)	0	0
England	3	<4	1643(9)	0.550(9)	5.64(4)	0	0
		<60	4213(18)	0.63(1)	5.18(3)	0	0
		5-9	1624(9)	0.55(1)	5.02(4)	0	0
		10-14	2410(14)	0.58(2)	4.36(4)	0	0

Continued on next page

Table S1– continued from previous page

Country	Wave #	age group	a	γ	t_0	% 1st dose	% 2nd dose
		15-19	3771(15)	0.630(9)	4.83(3)	0	0
		20-24	5344(21)	0.68(1)	5.22(3)	(18-24) 0.5	0.
		25-29	5629(24)	0.66(1)	5.18(3)	1.1	0.1
		30-34	5578(26)	0.63(1)	5.28(4)	1.2	0.1
		35-39	5289(25)	0.62(1)	5.25(4)	1.2	0.1
		40-44	5167(23)	0.62(1)	5.15(3)	1.5	0.1
		45-49	4769(20)	0.63(1)	5.15(3)	1.8	0.1
		50-54	4578(18)	0.638(9)	5.28(3)	2	0.1
		55-59	4147(17)	0.642(9)	5.36(3)	2.1	0.2
		60+	2779(9)	0.624(7)	5.46(3)	-	-
		60-64	3484(14)	0.641(9)	5.43(3)	1.8	0.1
		65-69	2348(10)	0.646(9)	5.38(3)	0.9	0.1
		70-74	1837(7)	0.644(8)	5.37(3)	0.7	0.
		75-79	2045(6)	0.619(6)	5.46(2)	1.9	0.1
		80-84	2654(7)	0.600(5)	5.48(2)	25.2	4.3
		85-89	4077(11)	0.582(5)	5.58(2)	26.6	3.4
		90+	6819(19)	0.585(5)	5.67(2)	22.9	3.7
	4	<4	1011(12)	0.570(6)	8.48(5)	0	0
		<60	3580(59)	0.610(9)	8.48(5)	0	0
		5-9	2099(18)	0.652(6)	7.83(4)	0	0
		10-14	4481(56)	0.629(8)	8.02(5)	0	0
		15-19	6543(80)	0.620(9)	8.02(5)	0	0
		20-24	6673(61)	0.642(7)	7.57(4)	(18-24) 61.8	20.9
		25-29	5452(137)	0.62(2)	8.3(1)	60.9	27.1
		30-34	4493(156)	0.60(2)	8.7(2)	64.1	40.6
		35-39	3529(109)	0.60(2)	8.6(2)	69.2	52.3
		40-44	3059(71)	0.60(2)	8.49(9)	75.4	66.6
		45-49	2456(53)	0.59(1)	8.49(9)	81.3	75.1
		50-54	2086(59)	0.58(2)	8.7(2)	86	82.4
		55-59	1659(53)	0.58(2)	8.9(2)	88.5	85.3
		60+	818(31)	0.56(2)	9.4(2)	-	-
		60-64	1277(51)	0.57(2)	9.2(2)	90.4	87.7
		65-69	898(36)	0.58(2)	9.4(2)	92.3	90.7
		70-74	627(20)	0.59(2)	9.2(2)	94.5	93.3
		75-89	566(16)	0.569(8)	9.4(1)	95.5	94.4
		80-84	584(21)	0.510(8)	10.0(2)	95.6	93.9
		85-89	686(33)	0.468(7)	10.7(2)	95.6	93.7
		90+	663(23)	0.495(7)	10.2(2)	94	91.8
